# Minilungs from Human Embryonic Stem Cells to Study the Interaction of Streptococcus pneumoniae with the Respiratory Tract

**DOI:** 10.1128/spectrum.00453-22

**Published:** 2022-06-13

**Authors:** Julio Sempere, Suélen Andreia Rossi, Irene Chamorro-Herrero, Fernando González-Camacho, María Pilar de Lucas, José María Rojas-Cabañeros, Carlos Pelleschi Taborda, Óscar Zaragoza, José Yuste, Alberto Zambrano

**Affiliations:** a Biotechnology of Stem Cells and Organoids, Chronic Diseases Program, Instituto de Salud Carlos III, Madrid, Spain; b Spanish Pneumococcal Reference Laboratory, Centro Nacional de Microbiología, and CIBER of Respiratory Diseases (CIBERES), Instituto de Salud Carlos III, Madrid, Spain; c Cellular Biology Unit, Chronic Diseases Program and CIBER of Cancer (CIBERONC), Instituto de Salud Carlos III, Madrid, Spain; d Department of Microbiology, Biomedical Sciences Institute, University of São Paulo (USP), São Paulo, Brazil; e Mycology Reference Laboratory, Centro Nacional de Microbiología and CIBER of Infectious Diseases (CIBERINFEC), Instituto de Salud Carlos III, Madrid, Spain; Geisel School of Medicine at Dartmouth

**Keywords:** minilungs, human pluripotent stem cells, human embryonic stem cells, hESCs, *Streptococcus pneumoniae*, pneumococcus, surfactant proteins, alveolar cells, disease modeling, biosurfactants

## Abstract

The new generation of organoids derived from human pluripotent stem cells holds a promising strategy for modeling host-bacteria interaction studies. Organoids recapitulate the composition, diversity of cell types, and, to some extent, the functional features of the native organ. We generated lung bud organoids derived from human embryonic stem cells to study the interaction of Streptococcus pneumoniae (pneumococcus) with the alveolar epithelium. Invasive pneumococcal disease is an important health problem that may occur as a result of the spread of pneumococcus from the lower respiratory tract to sterile sites. We show here an efficient experimental approach to model the main events of the pneumococcal infection that occur in the human lung, exploring bacterial adherence to the epithelium and internalization and triggering an innate response that includes the interaction with surfactant and the expression of representative cytokines and chemokines. Thus, this model, based on human minilungs, can be used to study pneumococcal virulence factors and the pathogenesis of different serotypes, and it will allow therapeutic interventions in a reliable human context.

**IMPORTANCE** Streptococcus pneumoniae is responsible for high morbidity and mortalities rates worldwide, affecting mainly children and adults older than 65 years. Pneumococcus is also the most common etiologic agent of bacterial pneumonia and nonepidemic meningitis, and it is a frequent cause of bacterial sepsis. Although the introduction of pneumococcal vaccines has decreased the burden of pneumococcal disease, the rise of antibiotic-resistant strains and nonvaccine types by serotype replacement is worrisome. To study the biology of pneumococcus and to establish a reliable human model for pneumococcal pathogenesis, we generated human minilungs from embryonic stem cells. The results show that these organoids can be used to model some events occurring during the interaction of pneumococcus with the lung, such as adherence, internalization, and the initial alveolar innate response. This model also represents a great alternative for studying virulence factors involved in pneumonia, drug screening, and other therapeutic interventions.

## OBSERVATION

Streptococcus pneumoniae (pneumococcus) is the leading cause of severe bacterial pneumonia, with high morbidity and fatality rates worldwide. Lower-respiratory infections were responsible for over 2.74 million deaths in 2015 and the third major cause of mortality worldwide in children under 5 years of age, with S. pneumoniae being the leading cause of these infections (1). During the last decade, interest in characterizing the complex interplay between host-microbe interactions using organoid cultures has increased. Organoids share important features with the original organ that make them attractive models for studying pathogenesis or microbial colonization of the tissue—for instance, the variety of cell types, the spatial arrangement of cells, and some level of organ functionality. A plethora of examples of organoids derived from adult stem cells and pluripotent stem cells (PSCs) to study the pathogenesis process and the interaction of microbial pathogens with the epithelium have been reported. Hence, organoids from the human liver, stomach, intestine/colon, and gallbladder have been used to explore infections by protozoan parasites and bacteria such as Escherichia coli, Helicobacter pylori, Clostridioides difficile, and Salmonella enterica serovar Typhimurium (2). The natural biology of pneumococcal interactions in the respiratory tract includes nasopharyngeal colonization, the production of acute otitis media, sinusitis and, in many cases, dissemination to the lower respiratory tract, producing pneumonia. The pneumococcal carrier state is usually an asymptomatic event for the vast majority of individuals, and it is a prerequisite for infection of the lower respiratory tract, producing pneumonia, bacteremia, and eventually meningitis. During the early stages of pneumonia, pneumococcus avoids the mucociliary and antibacterial peptide barriers by expressing different virulence factors (3). Adhesion to lung cells involves interactions with host cell glycoconjugates, followed by interaction with host cell protein receptors that promote internalization (4). As the alveolar epithelium is denuded, the underlying extracellular matrix of the alveoli is exposed; therefore, the interaction of S. pneumoniae with the alveoli results in inflammation and cytotoxicity, including accumulation of edema fluids, erythrocytes, and fibrins. At the point where alveoli are overfilled with bacteria, systemic dissemination could eventually take place by damaging the epithelium and directly invading endothelial cells. While knowledge about these processes is abundant, little is known about the specific interaction of pneumococcus with alveolar type I and II (ATI and ATII) cells (responsible for gas exchange and surfactant production) or their responses during the initial infection, including the early phases of acute pneumonia. This is partially due to the lack of reliable cell models and the difficulty of extracting, establishing, and maintaining primary ATI and ATII cells in culture. In the alveoli, ATII cells represent the preferred target for pneumococcal adherence and cell damage (5–8). An important defense system of the lung is the surfactant that protects the lung from injuries and infections and reduces the surface tension, thus preventing alveolar collapse at the end of expiration. The surfactant is a mixture of phospholipids, proteins, and carbohydrates that is produced and secreted by the ATII cells. Surfactant protein A (SFTPA) has an opsonizing activity that mediates phagocytosis and the removal of several respiratory microorganisms, including S. pneumoniae (9, 10). SFTPB peptides seem to mediate the aggregation of both Gram-positive and Gram-negative bacteria, contributing to their elimination by permeabilizing the bacterial cell membrane. SFTPD binds polysaccharides located on the bacterial surface and promotes aggregation, phagocytosis, complement activation, and the inhibition of bacterial colonization and invasion (11–15).

**FIG 1:**
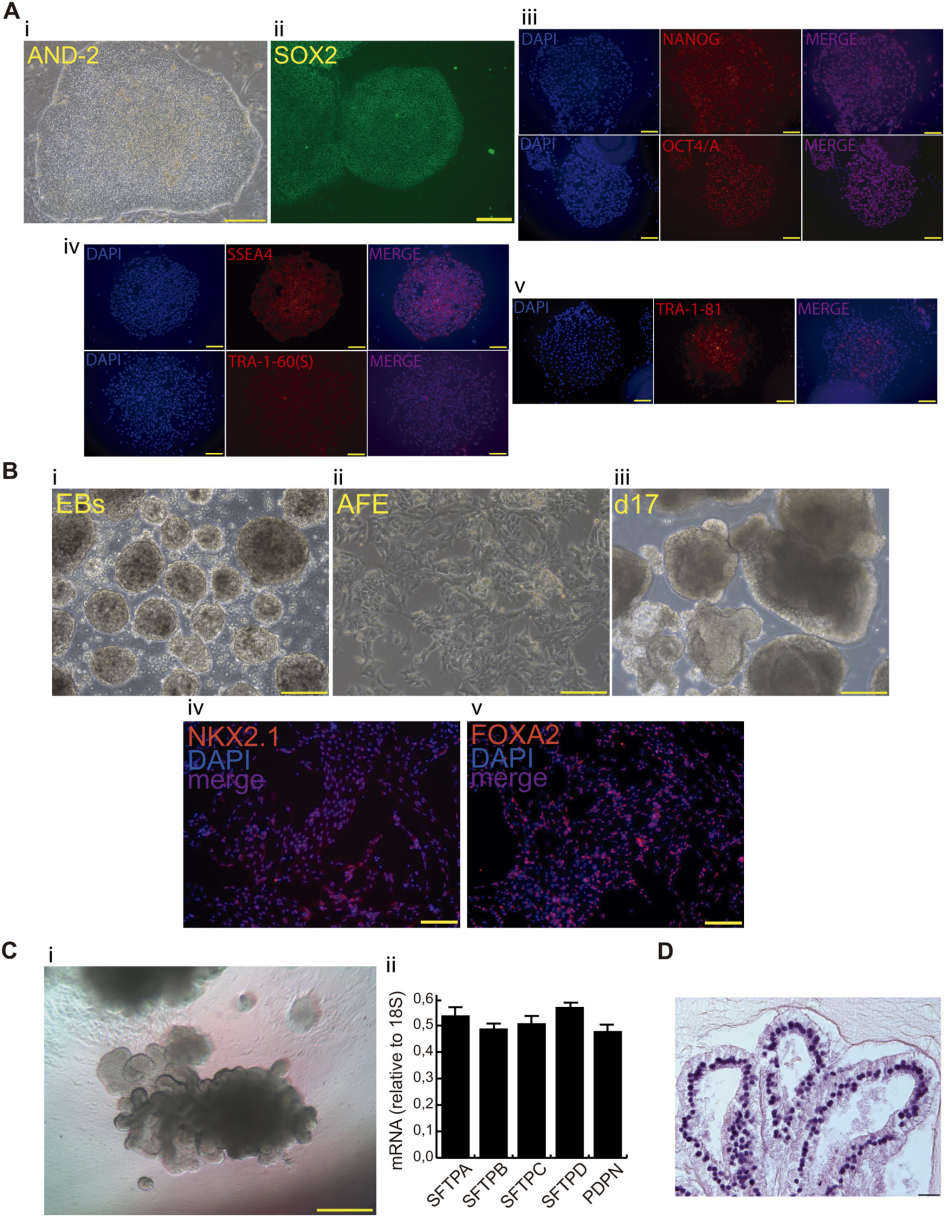
Generation of human minilungs: sequential differentiation process and expression markers. (Ai) AND-2 colony growing along with feeder cells (iMEFs); scale bar, 100 μm; (Aii) expression of SOX2 in an undifferentiated colony of AND-2; scale bar, 100 μm; (Aiii to Av) representative micrographs of pluripotency markers detected in AND-2 colonies (NANOG, OCT4/A, SSE4-A, TRA1-60[S], and TRA1-81); scale bar, 100 μm. (Bi to Biii) Representative micrographs of embryoid bodies (EBs), anterior foregut endoderm (AFE), and cultures at day 17 (d17) of differentiation (nascent organoids); scale bar, 100 μm. (Biv and Bv) Detection of FOXA2 and NKX2-1 (markers of the lung field) by indirect immunofluorescence. Negative controls of these immunofluorescences can be found in Fig. S1A and B in the supplemental material. (Ci) Representative micrograph of lung bud organoids embedded in Matrigel sandwiches and (Cii) expression levels (relative to 18S) of alveolar epithelial cells markers (day 56) (*n* = 3; >4 organoids per experiment were used; analysis of variance [ANOVA], *P* = 0.0674); scale bar, 100 μm. (D) Histochemical analysis of LBO sections (hematoxylin and eosin [H&E] staining) of organoids at day 60; scale bar, 50 μm.

**FIG 2:**
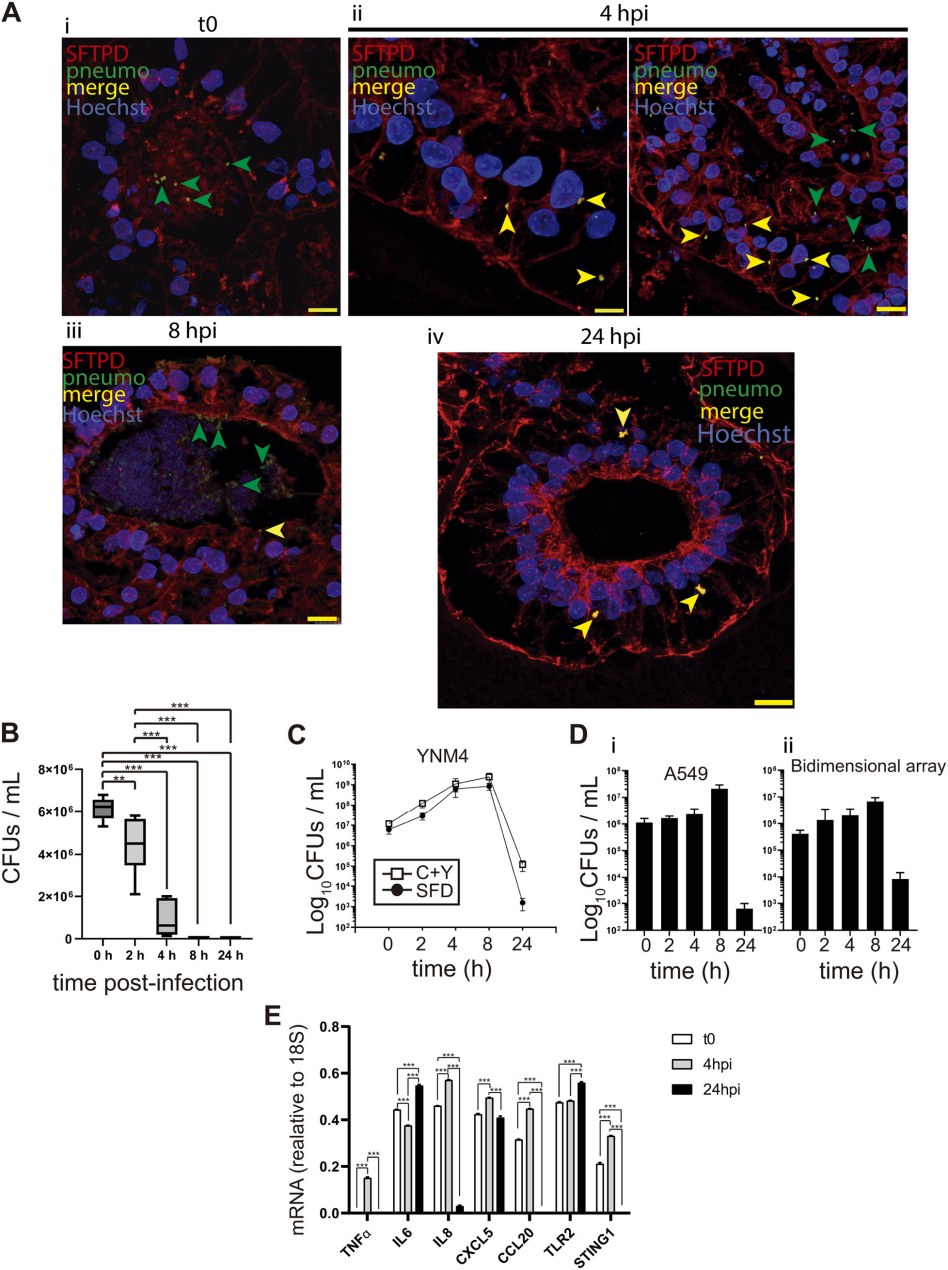
Interaction of pneumococcus with the surfactant system of the microinjected organoids. (A) Interaction of pneumococcus with lung buds at different times postmicroinjection (*t*_0_ to 24 h postinfection [hpi]). (Ai) Arrowheads signal pneumococcus particles at the lumen of the organoid; (Aii) green and yellow arrowheads signal pneumococcus particles at the lumen of the organoid and inside the alveolar epithelium (invaded epithelium), respectively; (Aiii) green and yellow arrowheads signal pneumococcus aggregates at the lumen of the organoid and inside the alveolar epithelium, respectively; (Aiv) yellow arrowheads signal pneumococcus and SFTPD aggregates inside the alveolar epithelium. (B) Quantification of CFUs/mL (*n* = 3; >4 organoids per condition were used; ANOVA, *P* < 0.0001). (C) Growth of strain YNM4 (S19A) in serum-free differentiation (SFD) and C+Y medium. (D) Dynamics of pneumococcal infection using A549 cells (Di) and bidimensional arrays of airway and lung epithelial cells derived from hESCs (Dii), (Di) and infected with strain YNM4. (E) RT-qPCR result of markers representing the alveolar innate response to pneumococcus infection at different times postinfection. *IL-6* (interleukin 6), *IL-8* (C-X-C motif chemokine ligand 8), *TNF-α* (tumor necrosis factor alpha), *TLR2* (Toll-like receptor 2), *STING1* (stimulator of interferon response cGAMP interactor 1), *CXCL5* (C-X-C motif chemokine ligand 5), *CCL20* (C-C motif chemokine ligand 20) (*n* = 3, >4 organoids per experiment were used; ANOVA, *P < *0.0001). The results presented are means ± SEM. The significance of the analysis is indicated as follows: *, *P < *0.05; **, *P < *0.01; ***, *P < *0.001.

We report here the characterization of a human organoid model suitable for studying pneumococcal interactions with the human alveolar epithelium. We used human embryonic stem cells (hESCs; AND-2 line) to build lung bud organoids (LBOs) that develop branching airway and early alveolar structures embedded in Matrigel sandwiches. The use of the hESC line AND-2 and the experimental procedures of this study were approved by the ISCIII Ethics Committee (reference no. CEI PI 10_2015-v2) and the National Committee of Guarantees for the Use and Donation of Human Cells and Tissues (reference no. 345 288 1 and 436 351 1). Lung organoids like those generated here have numerous advantages over cell lines or simple primary cultures, as they offer an unlimited availability of primary cells and can emulate structural and functional features of the original organ. The generation of optimal lung organoids relies primarily on the selection of good hESCs showing adequate morphology when growing, along with inactivated feeder cells (inactivated mouse embryo fibroblasts [iMEFs]). hESCs are picked up and passaged to new plates with iMEFs to accumulate material for subsequent differentiation to the lung lineage. Figure 1A shows the aspects of a good hESC colony, the expression of pluripotency markers (SOX2 [sex-determining region Y box 2], NANOG [Nanog homeobox], OCT4/A [*POU5F1*, POU class 5 homeobox 1], SSEA4 [stage-specific embryonic antigen-4], etc.). Figure 1B shows representative micrographs at various points of the differentiation process (embryoid bodies [EBs], anterior foregut endoderm [AFE], and cultures at day 17, representing nascent organoids in suspension) and the expression of the lung field specification markers NKX2-1 (NK2 homeobox 1) and FOXA2 (forkhead box A2) (at differentiation day 22) by indirect immunofluorescence. As previously reported, these two markers show a pan-nuclear homogeneous expression in nearly the whole cell population, indicating the correct differentiation to the lung field; negative controls are shown in Fig. S1A and B in the supplemental material. Figure 1C shows the microscopic aspect at day 56 of LBOs embedded in Matrigel sandwiches undergoing an adequate state of differentiation, characterized by the presence of lung buds more or less branched, as previously described (16, 17). Figure 1C also shows the results of reverse transcription-quantitative PCR (RT-qPCR), illustrating the complexity of these cultures that express significant levels of alveolar epithelial cell markers: PDPN (podoplanin; ATI cells) and the surfactants (ATII cells). As previously described by our group and others (16–20), the differentiation protocol applied here yields cultures enriched in alveolar epithelial cells, although specific markers of other cells (airway epithelial cells) were also found in proximal locations and after long-term maintenance of these structures (16) (Fig. S2). Figure 1D shows a representative micrograph of a histochemical analysis (H&E staining), denoting the typical branches of the LBOs at day 60. We performed microinjections of LBO branches at different locations to ensure the optimal delivery of pneumococci into the lumen, as we previously described for recombinant adenovirus (21). The internal connection of the branches allows spreading of the bacteria from the injection point to other sites. Further characterization of the LBOs generated included the expression of surfactant proteins and the proper visualization of pneumococcal cells by confocal microscopy (Fig. S1C and D). The expression of the surfactant proteins illustrates the maturity level of the organoid and the bias to ATII cells, a preeminent pneumococcal target in the alveoli. Additional markers for ATI and ATII cells (PDPN and ATP-binding cassette subfamily A member 3 [ABCA3], respectively), basal cells (keratin 5 [KRT5]), and ciliated cells (FOXJ1) are shown in Fig. S2A. In addition, we show the negligible expression of VIMENTIN (VIM; mesenchymal cells) in the minilungs generated (Fig. S2A). Pneumococcal cells were easily observed in the lumen of the branches, attached at the cell surface, or immersed in the cytoplasm of the cells, delineating the distal branches of the organoid. Analysis by confocal microscopy indicated an unequivocal interaction between pneumococcus and surfactant proteins (Fig. S1C). To model pneumococcal infection within the alveolar epithelium, we delivered pneumococcal cells into the lumen of the organoids and followed the interaction, in this case, with SFTPD, at different times. The microinjected organoids were processed for immunofluorescence, and colony counts were quantified at different postinfection times (2, 4, 8, and 24 h). Figure 2A illustrates the transit of the bacteria through the organoid epithelium, demonstrating the proper delivery of the bacteria to the lumen, adherence to the ATII cell surface, and internalization. At *t*_0_, there were multiple bacteria in the lumen (green staining; Fig. 2Ai). At 4 and 8 h, we observed the presence of numerous pneumococcal cells in the lumen (green staining; green arrowheads), interacting with SFTPD at different locations and infecting the cytoplasm of the ATII cells (merged staining; yellow arrowheads) (Figure 2Aii, iii). Figure 2Aiv shows a significant decrease in the number of pneumococci (green staining) inside the ATII cells and their absence in the LBO lumen, indicating, very likely, that surfactant could be contributing to the clearance of bacterial CFUs (colony-forming units). In addition, we observed the presence of few but particularly large pneumococcus-SFTPD aggregates inside the cell (Fig. 2Aiv, merged staining; yellow arrowheads). The dynamics delineated by this microscopic analysis fit well with the numbers of CFUs found in extracts of the microinjected organoids at different times postinfection. Hence, at *t*_0_ (organoids processed immediately after the microinjection), CFUs on the order of 6 × 10^6^ were found in the cell extracts (Fig. 2B). A fast decay in the number of viable bacteria was observed between 2 h and 4 h and from 4 h to 8/24 h. The lack of viable bacterial counts in the extracts is related to the absence of pneumococcal cells in the immunofluorescence (green staining) and the presence of those large pneumococcus-SFTPD aggregates observed at 24 h (Fig. 2A). The bacterial strain used in this work, YNM4, shows similar growth rates in C+Y medium (CpH8 medium with 0.08% yeast extract) and in the serum differentiation medium (SFD) employed in the differentiation process (Fig. 2C). The bacterium also shows an equivalent viability when exposed to A549 cells and to airway and lung epithelial cells, displayed as a bidimensional array (generated as described previously [18]) (Fig. 2D). Similar results were obtained with strain D39, which is a different serotype of S. pneumoniae (Fig. S3). To evaluate the host-pathogen interactions, we used bacterial mutants in LytA, PspC, and the double mutant in LytA/PspC, because they are important virulence factors involved in pathogenesis (22, 23). Our results confirm that strains lacking LytA (the major autolysin of the bacterium) can grow for longer periods in both kinds of media, confirming that the composition of the SFD medium does not affect the normal physiology of the autolytic system (Fig. S3D). Moreover, YNM4 showed growth in viability up to 8 h in the attachment to A549 cells and in the airway and lung epithelial cells, whereas viability in lung organoids decreased after *t*_0_. Hence, the decay in 3D organoids may reflect the potential contribution of surfactant to the control of bacterial CFUs and not an increase in the autolysis influenced by the medium used. In addition, infection of epithelial cells from the bidimensional array and A549 cells with strains lacking these proteins (especially PspC) showed reduced bacterial levels compared to the wild-type strains. These results demonstrate that LytA and PspC, to a greater extent, are surface-exposed proteins involved in the interaction of S. pneumoniae with lung epithelial cells (Fig. S3). Taken together, these results suggest that the fast decay of bacterial viability observed might be due to the interaction with surfactant that could be contributing to the control of bacterial levels. It has been reported that different surfactant proteins, such as SFTPA and SFTPB, have antimicrobial activity by affecting the depolarization and permeabilization of the bacterial membrane and the induction of toroidal pores (13, 24). The early interaction of the surfactant with the bacteria described here correlated with the innate response of the alveolar cells to the pneumococcal infection. ATII cells and, to a lesser extent, ATI cells are essential effector cells in the inflammatory response to pneumococcal infection, even when they cover around 5% of the alveolus surface (25). Upon infection, ATII cells can release a plethora of antimicrobial molecules, cytokines, and chemokines (25, 26). These mediators contribute to the migration of monocytes and macrophages to the site of infection and their activation. Figure 2E shows the RT-qPCR result corresponding to different components of the innate response induced by the organoids infected with pneumococcus. We analyzed *TLR2*, *IL-6* (interleukin 6), *IL-8*, *TNF-α*, *CXCL5*, and *CCL20* (*MIP3*) because they are important components of the host immune response involved in the recognition of and inflammatory response to invading pathogens such as S. pneumoniae (7, 8, 26–28). Our results indicate a rapid response of the alveolar epithelium upon exposure to S. pneumoniae. We also observed that pneumococcal infection of the lung organoids triggered the expression of many of these mediators, reaching a peak at 4 h, and *TLR2*, showing an upward trend of expression. This is consistent with previous data showing the relevance of TLR2, which is expressed on the surface of ATII cells, and it has been shown to allow NF-κB activation after pneumococcal infection (29). In addition, we show the expression of TLR2, TLR9, and PAFR (GPR135, G protein-coupled receptor 135 [PAF receptor]) in the organoids of this study, indicating the suitability of the minilungs as a platform for evaluating cellular innate responses and important virulence factors (Fig. S2B).

Overall, our study demonstrates the suitability of hESC-derived lung organoids for modeling early interactions of S. pneumoniae with the alveolar epithelium, including the host immune response. Below, we summarize the potential of the model presented here in terms of human material availability, time, and relevance to pneumococcal biology.

1. These minilungs are human primary cells organized into 3D structures mimicking the structure and function of the native organ.
2. The system shows an unlimited and rapid availability of precious primary human material. The hESCs or lung lineage progenitors can be expanded to an unlimited extent to get enough material for the differentiation process.
3. Infective bacteria can be delivered directly to the lumen by microinjection to mimic alveolus exposure to exogenous agents.
4. This model has enormous potential for studying the adherence of bacteria to the eukaryotic cell during the early stages and the influence of bacterial virulence factors. This potential includes the possibility of therapeutic interventions: developing antiadhesive strategies to block transmission and colonization.
5. This model will allow the establishment of specific individual organoids, organoid biobanks, and high-throughput analyses. The application of this protocol to induced PSCs (iPSCs), for instance, will allow the development of individual-specific minilungs to study pneumococcal interactions with the alveolar epithelium, alveolar innate responses, and the interaction of pneumococcus with chronic diseases such as chronic obstructive pulmonary disease (COPD). In addition, the use of human PSCs (hPSCs) may serve to set up biobanks with minilungs from different individuals for modeling targeted drug treatments.

### Data availability.

Please contact the corresponding author for data requests.
