# Supplementary material for: Minilungs from Human Embryonic Stem Cells to Study the Interaction of Streptococcus pneumoniae with the Respiratory Tract
Source: Microbiol Spectr. 2022 Jun 13;10(3):e00453-22. doi: 10.1128/spectrum.00453-22 (PMC9241785; doi:10.1128/spectrum.00453-22)
Supplement: Supplemental file 1 — Supplemental material. Download spectrum.00453-22-s0001.pdf, PDF file, 4.6 MB [file spectrum.00453-22-s0001.pdf]

Supplemental Figure 1 Sempere et al.

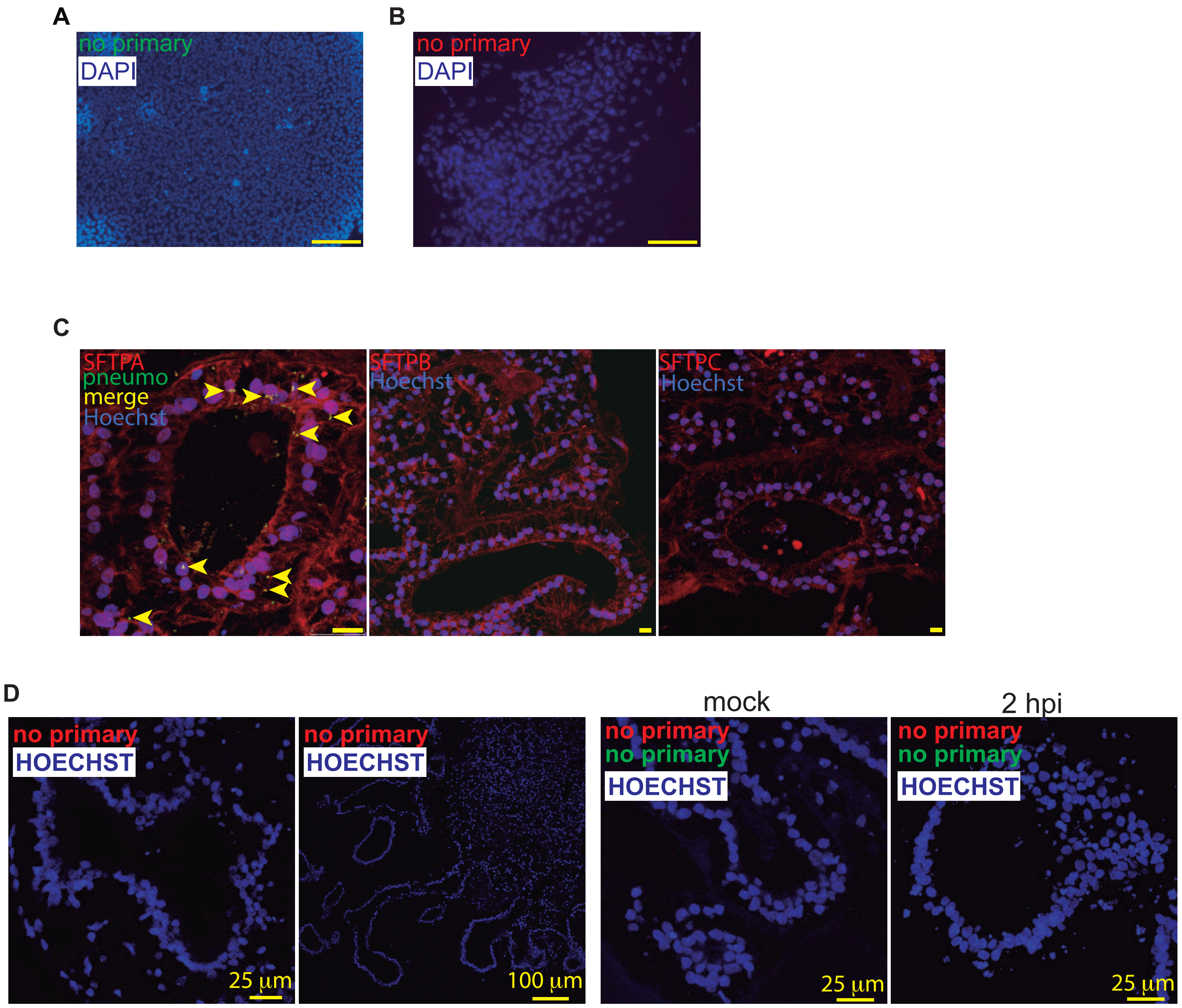

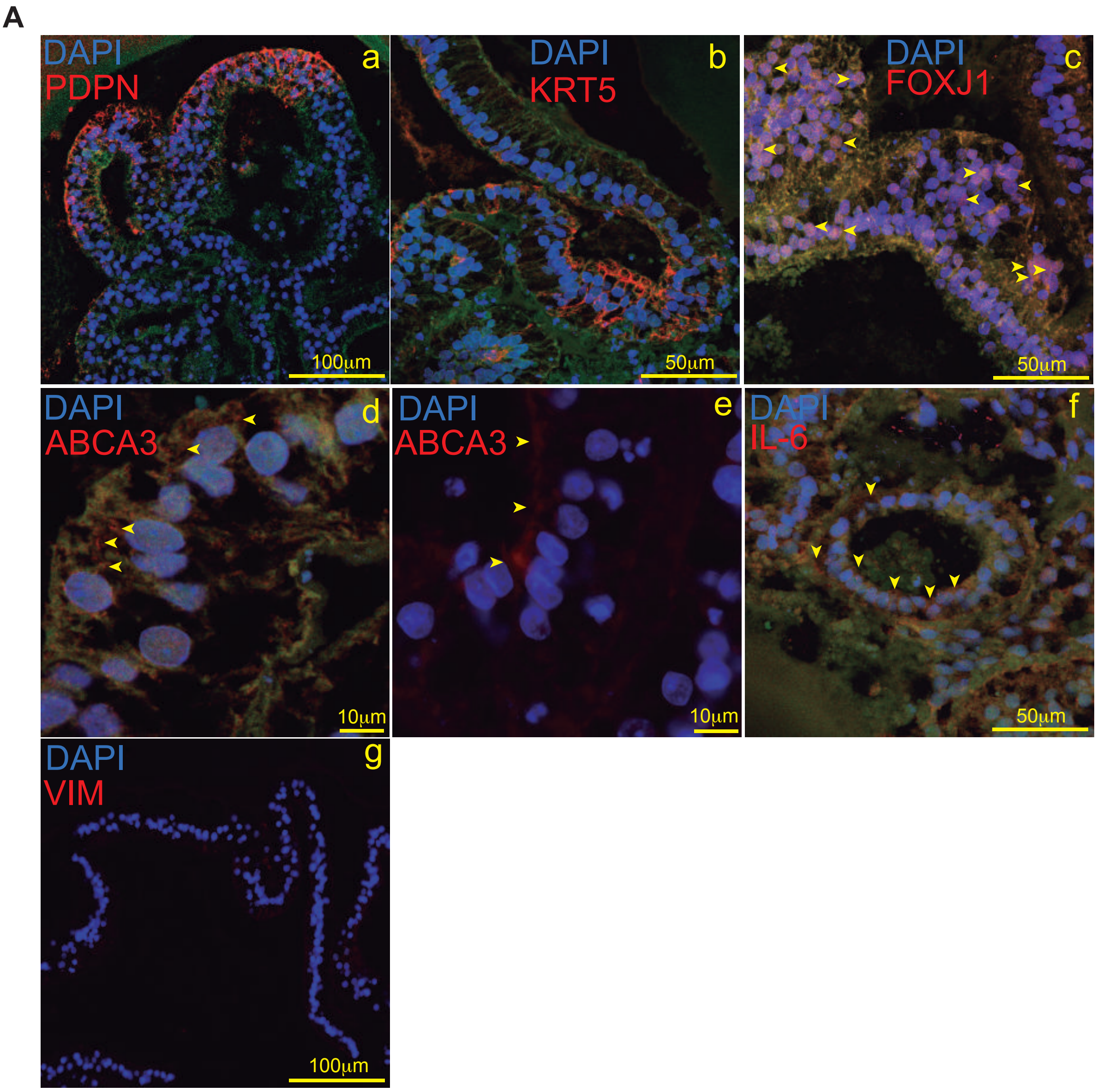
A
DAPI
PDPN
a
100µm
DAPI
KRT5
b
50µm
DAPI
FOXJ1
c
50µm
DAPI
ABCA3
d
10µm
DAPI
ABCA3
e
10µm
DAPI
IL-6
f
50µm
DAPI
VIM
g
100µm

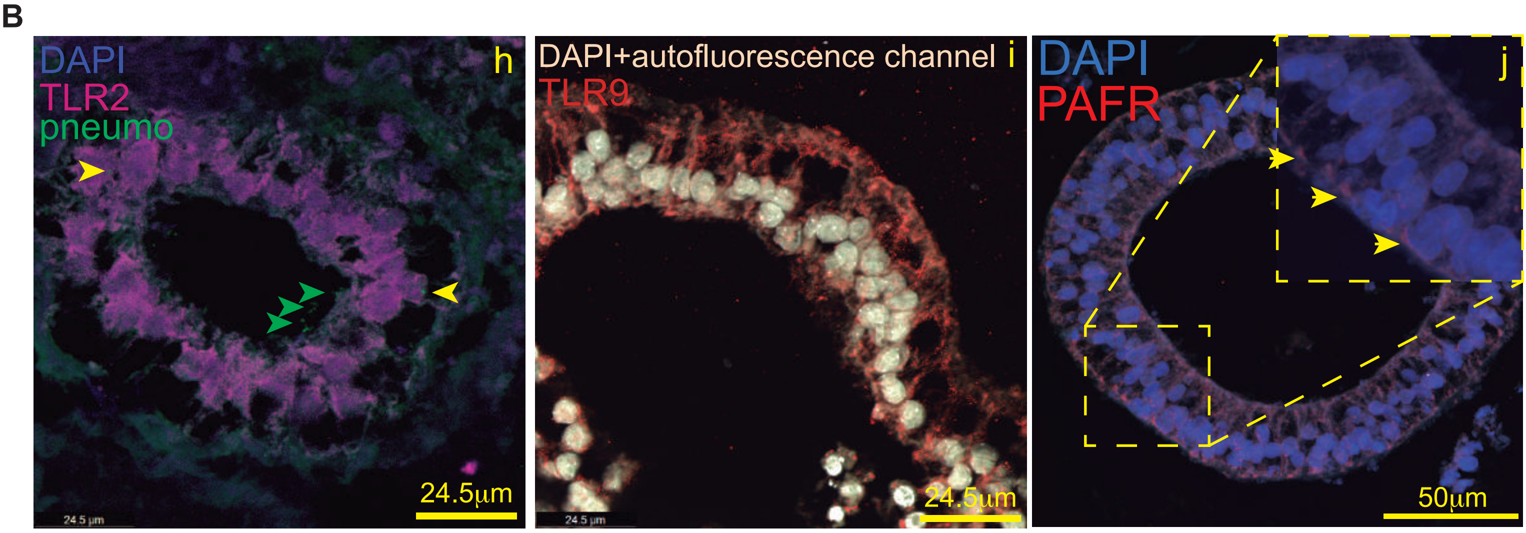
B
DAPI
TLR2
pneumo
h
24.5µm
DAPI+autofluorescence channel
TLR9
i
24.5µm
DAPI
PAFR
j
50µm

Supplemental Figure 3   Sempere et al.

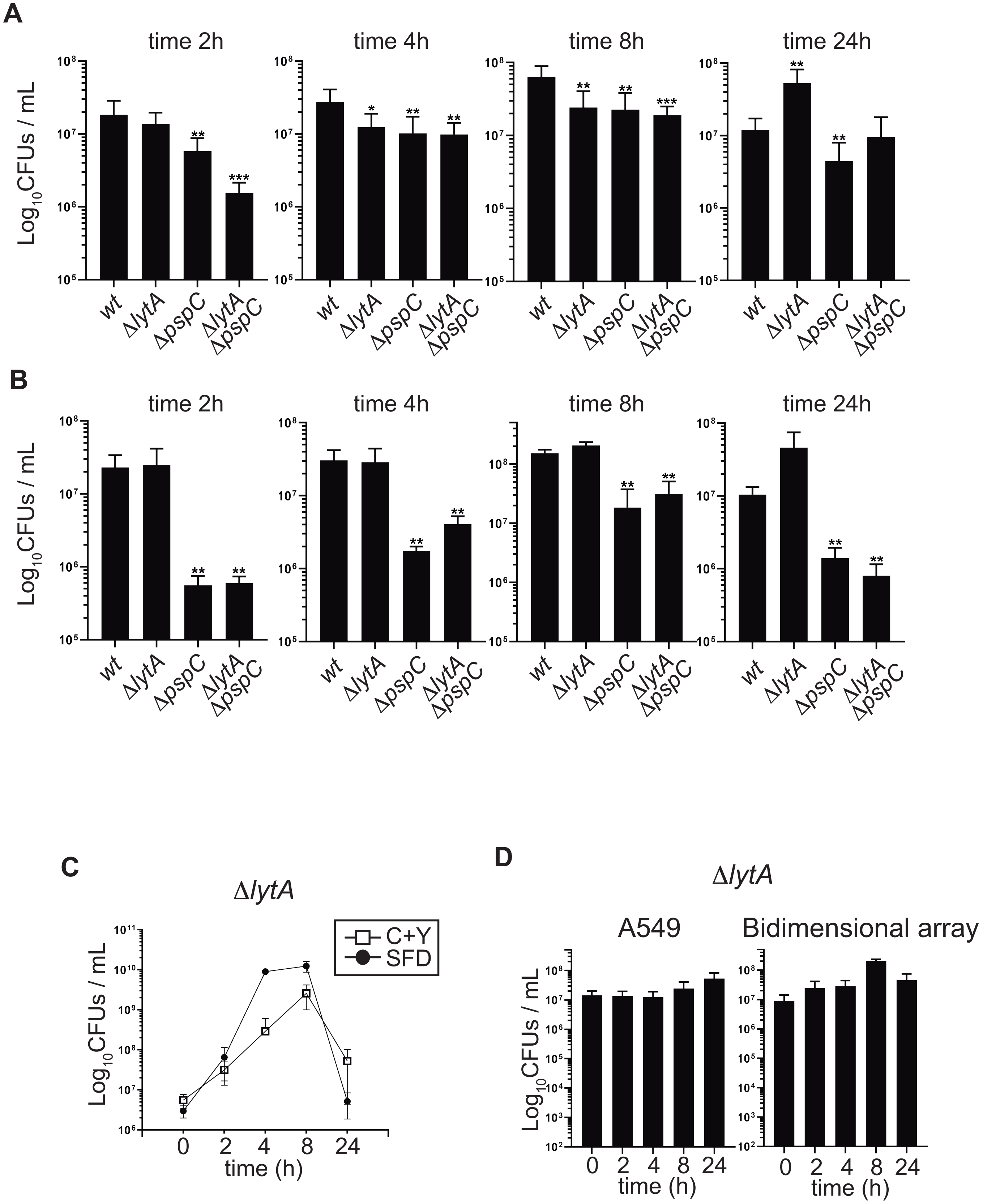

Supplemental Figure 1. Representative micrographs of negative controls (no primary controls). **A.** No primary controls for the immunofluorescences of the pluripotency markers. **B.** No primary controls for NKX2-1 and FOXA2 immunofluorescences. Scale bar: 100 μm. **C.** Representative micrographs of indirect immunofluorescences (confocal microscopy) of SFTPA, SFTPB and SFTPC of the lung organoids; scale bar: 10 μm. Arrowheads signal pneumococci and SFTPA colocalization. **D.** Representative micrographs of negative controls (no primary controls) for the surfactant proteins and pneumococcus immunofluorescences.

Supplemental Figure 2. Expression of cellular markers and host cell receptors on organoids **A.** Representative micrographs of markers of airway and alveolar cells, mesenchymal cells and interleukin 6; **a:** Podoplanin (PDPN, marker of ATI cells); **b:** KRT5 (basal cells); **c:** FOXJ1 (ciliated cells); **d,e:** ABCA3 (lamellar bodies, mature ATII cells); **f:** Interleukin 6 (IL-6) expression (in an infected organoid, 24 hpi); **g:** VIM (vimentin, mesenchymal cells). **B.** Representative micrographs of the expression of TLR2 (in an infected organoid, 24 hpi), TLR9 and PAF receptor (PAFR).

Supplemental Figure 3. Dynamics of growth and infection of *S. pneumoniae* mutant strains. **A.** Infection of A549 cells using D39 strains and isogenic mutants *lytA, pspC* and the double mutant *lytA pspC*. **B.** Infection airway and lung epithelial cells derived from hESCs (bidimensional array) using D39 strain and isogenic mutants *lytA, pspC* and the double mutant *lytA pspC*. **C.** Growth of D39 *lytA* strain in SFD or C+Y medium. **D.** Dynamic of infection of a D39 *lytA* deficient strain in A549 cells (left panel) or epithelial cells derived from hESCs (right panel). The results presented in the figures are means ±

SD. Significance of the analysis is indicated as, *: $P<0.05$, **: $P<0.01$, ***: $P<0.001$.

Supplemental material

MATERIAL AND METHODS:

Maintenance of hESCs

The hESCs line AND-2 was obtained from the Stem Cells Biobank at Granada (ISCIII, Spain); passages 26-40. Mouse embryonic fibroblasts (MEFs) were obtained at 13.5 days post-coitum from C57BL/6 mice as described previously (1). MEFs were mitotically inactivated by overnight treatment with 2 µg/mL of mitomycin C (cat.#M4287; Sigma-Aldrich) and plated at a density of approximately 16,000 cells/cm^2. hESCs were cultured along with MEFs under standard conditions (2). The maintenance medium was composed of KO-DMEM (Gibco; Life Technologies), 20% KO serum replacement (Gibco; Life Technologies), 0.1 mM β-mercaptoethanol (cat.#21985-023 Gibco; Life Technologies), 2 mM Glutamax (cat.#35050-061,Gibco; Life Technologies), non-essential amino acids (cat.#11140-050 Gibco; Life Technologies and primocin (cat.#12I05MM; InvivoGen). The medium was filtered by using 0.22-µ pore filter systems (cat.#431097; Corning); 10 ng/mL recombinant human basic Fibroblast Growth Factor (hbFGF, FGF2) (cat.#PHG6015; Invitrogen) and 10 µM Y-27632 (cat.#1254; Tocris R&D Systems) were added before use. The medium was changed on a daily basis and cells were passaged either by enzymatic (collagenase IV method) (collagenase IV: cat.#11140050; Gibco; Life Technologies) or mechanical procedures (2). Cells were maintained in an undifferentiated state in a 5% CO_2/air environment. The differentiation process was carried out in a 5% CO_2/5% O_2/95% N_2 environment [Galaxy 48R incubator (New Brunswick)], until the formation of anterior foregut endoderm (AFE).

Primitive streak formation and induction of definitive endoderm (DE)

Induction of endoderm was established as previously described (3) (4). Primitive streak formation (day 0; 24h) and endoderm induction (days 1-4) were performed in serum-free

differentiation (SFD) medium. SFD medium was composed of a mix of IMDM:F12 (3:1) media (cats.#B12-722F and 10-080 CVR; Corning), supplemented with N2 (cat.#17502-048, Gibco; Life Technologies), B27 (cat.#17504-044, Gibco; Life Technologies), 2 mM Glutamax (cat.#35050-061 Gibco; Life Technologies), 1% penicillin-streptomycin (DE17-602E; Lonza), and 0.05% bovine serum albumin (BSA) (cat.#A7906; Sigma-Aldrich). The medium was filtered using a 0.22 µ-pore filter system (cat.#431097; Corning); 50 µg/mL ascorbic acid (cat.#A4554; SigmaAldrich) and 0.04 µL/mL monothioglycerol (stock >97%) (cat.#M6145; Sigma-Aldrich) were added before use. MEFs were depleted by passaging hESCs lines onto Matrigel™-coated (cat.#354230; Life Technologies) plates for at least 24 h. Cells were briefly trypsinized into small 3-10 cell clumps and the reaction was halted with stop medium [IMDM medium (BE12722F) supplemented with 50% fetal bovine serum (F7524, Sigma-Aldrich), 2 mM Glutamax, 1% penicillin-streptomycin and 30 ng/mL DNase I (cat.#260913-10MU; Calbiochem)]. Cells were then centrifuged for 5 min at 138g and washed carefully two times with an excess of SFD medium. To generate embryoid bodies (EBs), the clumps were plated onto low-attachment 6-well plates (cat.#3471; Corning) and maintained in SFD medium in a 5% CO_2/5% O_2/95% N_2 environment (Galaxy 48R incubator; New Brunswick).

For primitive streak formation, 10 µM Y-27632, 10 ng/mL Wnt3a (cat.#5036-WN; R&D Systems) and 3 ng/mL human BMP4 (cat.#314-BP; R&D Systems) were used. EBs were collected, resuspended carefully in endoderm induction medium containing 10 µM Y-27632, 0.5 ng/mL human BMP4, 2.5 ng/mL hbFGF, and 100 ng/mL human Activin (cat.# 338-AC; R&D Systems). Cells were fed after 36–48 h, depending on cell density, by removing half the old medium and adding half fresh medium.

Induction of anterior foregut endoderm (AFE)

AFE (days 4, 5 or 5) was induced as previously described (3) (4). EBs were dissociated into single cells with trypsin and they were transferred to a conical tube containing a stop medium to neutralize trypsin. Cells were centrifuged for 5 min at 138g, washed carefully twice with SFD medium and counted. For AFE induction, 25,000-30,000 cells/cm^2 were plated on fibronectin-coated (F0895; Sigma-Aldrich) 12-well tissue culture plates in AFE induction medium 1 [SFD medium supplemented with 10 mM SB-431542 (cat.#1614; Tocris) and 100 ng/mL of NOGGIN (cat.#6057;, R&D Systems). After 24 h of incubation, the medium was aspirated and AFE induction medium 2 [SFD medium supplemented with 1 µM IWP2 (cat.#3533; Tocris) and 10 µM of SB-431542] was added to the cultures. This process was carried out under normoxy.

Formation of nascent lung bud organoids

On day 6.5-7, AFE cultures were treated for 2 days with the ventralization/branching medium (SFD medium supplemented with 3 µM CHIR99021 (cat.#04; Tocris), 10 ng/mL human FGF10 (cat.#345-FG; R&D Systems), 10 ng/mL human KGF (cat.#251KG-010; R&D Systems), 10 ng/mL human BMP4 (cat.#314-BP; R&D Systems) and 50 nM all-trans retinoic acid (cat.#R2625; Sigma-Aldrich). On day 8, cells were resuspended by gently pipetting up and down using p1000 pipette tips. The suspended cell clumps were transferred to ultra-low attachment MW6 plates (typically, one MW6 plate per two MW12 plates containing the AFE cultures). These clumps were fed every other day by tilting the plate and allowing the suspended clumps to sink to the bottom edge or transferring the cell clumps carefully to a sterile tube and centrifuging 1 min/ RT / 136g. When the clumps are becoming in nascent organoids showing the first signs of ventralization, the organoids were fed exclusively by tilting the plates.

Formation of lung bud organoids

The nascent organoids were maintained in a ventralization/branching medium for approximately 20-25 days. After that, these nascent organoids were embedded into Matrigel™ sandwiches assembled on MW24 wells. Briefly, 150 μL of Matrigel™ was loaded on the MW24 well and allowed to gel. Nascent organoids were picked up with a wide mouth plastic Pasteur pipette (high-performance transfer pipette, cat.#612-2857, VWR) or a manually cut p1000 pipette tip to get a wider end section. The good nascent organoids were divided into the MW24 wells containing approximately 50% Matrigel™ diluted in ventralization/branching media coming from the picked organoids and immediately transferred onto the first layer of Matrigel™. After solidification of this intermediate layer containing the nascent organoids, 150 μL of Matrigel™ was added on top. Finally, each sandwich containing various organoids was incubated with 600 μL branching media. The medium was changed completely or partially every 2–3 days. Growing branching structures were easily visualized under the microscope after 1 or 2 weeks. Treatments were performed in minilungs maintained in ventralization/branching medium under normoxic conditions. Two or three days before the microinjection of the bacteria, the sandwiches were washed with ventralization/branching medium lacking antibiotics and maintained under these conditions until the end of the experimentation.

Strain, media and microinjection

We used the *Streptococcus pneumoniae* YNM4 strain expressing a 19A capsular polysaccharide (M11 transformant with DNA from strain 1228/19; serotype 19A) (5). Bacteria were grown in a C medium supplemented with yeast extract (C+Y medium). Bacterial growth was monitored by measuring the absorbance at 550 nm (A_{550}). For microinjections, the bacterial stocks were centrifuged and resuspended with PBS to a concentration of 10^9 colony formation units (CFUs)/mL.

For microinjection, needles and the specific conditions for their construction and manipulation have been described in our previous work (6). The organoids were picked-up with the surrounding Matrigel^{TM} from the 24-well culture plate and transferred on a sterile microscope slide. Microinjections used the electronic FemtoJet microinjector, the Injectman Ni 2 micromanipulator (both Eppendorf) and Zeiss inverted microscope (Axio Observer 3). With the micromanipulator, the tip of the needle was maneuvered as close as possible above the organoid and when both the organoid and the needle tip shared the same focal plane, the needle was slowly lowered until it penetrated the organoid. The position of the needle inside the organoid was adjusted until the organoid visibly pulsated during injection, which was recorded as a successful injection. Depending on a visual assessment of the organoid size and number of buds, 2-3 locations were injected [300 to 600 nL; p_i 850 hPa, p_c 8 hPa, time 0.1 s) at the base of branches to reach an approximated MOI=10 according to a rough estimation of the number of cells present in the LBOs (6). After injection, the organoid was transferred with a Pasteur pipette (high-performance transfer pipette, cat.#612-2857, VWR) containing media to a 24-well plate with fresh SFD media without antibiotics. For CFUs assays, the organoids were mechanically homogenized in PBS. Then, we performed serial dilutions of the mixture that were plated on blood agar plates for bacterial counts determination. Plates were incubated for 24 h at 37°C with 5% CO_2. Results express viability as CFU/ml.

Indirect immunofluorescence (epifluorescence and confocal microscopy)

For the immunofluorescence of undifferentiated hESCs colonies or cells growing on human fibronectin, the cultures were fixed in 2% PFA in PBS for 10 min at RT and permeabilized with PBS-BSA 1%-0.25% Triton X-100 (5 min/RT). Preparations were then washed with PBS, blocked for 30 min with PBS/BSA1% and incubated overnight with antibodies against SOX2 (1:100; C-17 sc-17320, Santa Cruz Biotech.), additional

pluripotency markers (StemLieghtTM pluripotency kit, cat.# 9656, Cell Signaling), NKX2.1 (1:100; ab76013, Abcam) or FOXA2 (1:100; TA319660; Origene). Preparations were then washed with PBS and incubated with secondary antibodies conjugated with AF546, or AF488 (cats.#A11035, A11029, A11030, (Life Technologies); cat.#150129, Abcam) for 1 h at RT. Nuclei were counterstained with DAPI (300nM; Invitrogen cat.#D1306), and samples were mounted with ProLong Diamond (cat.#P36961, Life Technologies). Cell images were captured with fluorescence microscopy (Zeiss Axio) equipped with a camera (Axiocam MRm) and AxioVision software. For the immunofluorescence of LBOs: Microinjected organoids were transferred into small pore cassettes (0.30 mm^2) for microbiopsies (cat.# 100703, Labolan) and fixed overnight in buffered-formalin. After that, the cassettes were transferred to a container containing 70% ethanol and subjected to paraffin embedding and sectioning. 4μm-sections were used for the immunofluorescence protocol. Antigen retrieval and immunohistochemical staining were performed as previously described (1). After the antigen retrieval treatment, the paraffin-embedded sections were subjected to indirect immunofluorescence as described above. Nuclei were counterstained with Hoechst 33342 (cat.# 83218; AnaSpec Inc.) or DAPI (300nM; Invitrogen cat.#D1306) and ProLong gold antifade (cat.#P36934; Thermofisher Sci.) was used as mounting medium. The samples were subjected to confocal laser scanner microscopy (CLSM) using a Leica spectral TCS-SP5 confocal microscope equipped with a 40x oil immersion -lens objective (HC PL APO 40x/1.3 oil CS2), and a 63x oil immersion objective (HCX PL APO CS 63×/1.4). We performed up to 4x optical magnifications when needed. Excitation lines were 405-nm diode for Hoechst, 488-nm with an argon laser at 20% intensity for FITC and 561-nm diode for TRITC. Images were acquired and processed using LAS AF software (Leica Microsystems). For their analysis, the samples were scanned at 400 Hz and 1024x1024

PPI resolution. XYZ stacks (optical sections) were acquired frame by frame in sequential mode with a pinhole diameter at 1 AU (airy unit) and a z-step of 0.5 µm. The micrographs shown were obtained by single plane (an optical Z section), maximum projection or orthogonal projection. Primary antibodies used: sera against Pneumococcus [anti-serum pool B (SSI pool Serum B) and serum 19 (Group serum 19) (1:100; Statens Serum Institut, Copenhagen, Denmark)], SFTPA (1:200; clone MR334; cat.#MAB21977; Abnova), SFTPB (1:300; sc-133143 Cruz Biotech.), pro-surfactant protein C (1:200; ab3785, Merck), SFTPD (1:200; clone 2C10, cat.#H00006441, Abnova). ABCA3 (cat.# PA552478, Invitrogen and cat.# HPA007884, Sigma-Aldrich), PAFR (cat.# sc-20732), PDPN (cat.#ATGA0418, NKMAX)), KRT5 (cat.# orb213163, biorbyt), FOXJ1 (AMAb91254, Atlas Antibodies), IL-6 (cat.# AH1040, Bio-Rad), TLR2-647 (cat.# 15307, Biolegend), TLR9 (cat.# sc-25468). Secondary antibodies were used at 1:300 dilutions (cat.# 4030-02 and 1030-03, Southern Biotech; cats.# A11035, A11030, Life Technologies).

Quantitative real-time RT-PCR (RT-qPCR) of minilungs

Total RNA was extracted using RNAeasy kit from Qiagen (cat.#15596026) following the manufacturer's instructions. cDNA was generated using the High-Capacity cDNA kit (cat.#4387406; Applied Biosystems). Real-time qPCR was performed by using the power-up SYBR Green mix (cat.#A25742) on the Quantstudio-3 system (Applied Biosystems) following the manufacturer's instructions. Absolute quantification of each gene was obtained using a standard curve of serial diluted genomic DNA (cat.#11807720, Roche) and normalized to housekeeping gene 18S. The Supplemental Table shows the genes analyzed and the sequences of the oligonucleotides employed in this study. n=3 experiments, > 4 organoids per experiment were used.

Statistical analysis

Data were subjected to the Shapiro-Wilk test and D'Agostino and Pearson omnibus test to verify their normality. Statistical significance of data was determined by applying a two-tailed Student's t-test or an analysis of variance followed by the Tukey or Bonferroni post-tests for experiments with more than two experimental groups. $P < 0.05$ is considered significant. Significance of analysis of variance post-test or the Student's t-test is indicated in the figures as *, $P < 0.05$; **, $P < 0.01$; and ***, $P < 0.001$. Statistics were calculated with the Prism 9 software (GraphPad Software). The results presented in the figures are means ±SEM. Experiments were repeated three times.

Supplemental Material

SUPPLEMENTAL REFERENCES FOR MATERIALS AND METHODS

1. Zambrano A, García-Carpizo V, Gallardo ME, Villamuera R, Gómez-Ferrería MA, Pascual A, Buisine N, Sachs LM, Garesse R, Aranda A.. The thyroid hormone receptor β induces DNA damage and premature senescence.. J Cell Biol.. 2014 Jan 6; 204(1): p. 129-46.
2. http://www.stembook.org
3. Magro-Lopez E, Palmer C, Manso J, Liste I, Zambrano A. Effects of lung and airway epithelial maturation cocktail on the structure of lung bud organoids. Stem Cell Res Ther. 2018 Jul 11; 9(1): p. 186.
4. Magro-Lopez E, Guijarro T, Martinez I, Martin-Vicente M, Liste I, Zambrano A. A Two-Dimensional Human Minilung System (Model) for Respiratory Syncytial Virus Infections.. Viruses. 2017 Dec 10; 9(12): p. 379.
5. Sempere J, De Miguel S, Gonzalez-Camacho F, Yuste J, Domenech M. Clinical relevance and pathogenesis of the emerging serotypes 22F and 33F of *Streptococcus pneumoniae* in Spain. Front Microbiol. 2020 Feb 27;11:309.
6. Ramos-Sevillano E, Urzainqui A, Campuzano S, Moscoso M, González-Camacho F, Domenech M, Rodríguez de Córdoba S, Sánchez-Madrid F, Brown JS, García E, Yuste J. Pleiotropic effects of cell wall amidase LytA on Streptococcus pneumoniae sensitivity to the host immune response. Infect Immun. 2015 Feb;83(2):591-603.
7. Morey P, Cano V, Martí-Lliteras P, López-Gómez A, Regueiro V, Saus C, Bengoechea JA, Garmendia J. Evidence for a non-replicative intracellular stage of nontypable Haemophilus influenzae in epithelial cells. Microbiology (Reading). 2011 Jan;157(Pt 1):234-250.
8. Hendriksen WT, Kloosterman TG, Bootsma HJ, Estevão S, de Groot R, Kuipers OP, Hermans PW. Site-specific contributions of glutamine-dependent regulator GlnR and GlnR-regulated genes to virulence of Streptococcus pneumoniae. Infect Immun. 2008 Mar;76(3):1230-8. doi: 10.1128/IAI.01004-07. Epub 2008 Jan 3. PMID: 18174343; PMCID: PMC2258823.
9. Meyer-Berg H, Zhou Yang L, Pilar de Lucas M, Zambrano A, Hyde SC, Gill DR. Identification of AAV serotypes for lung gene therapy in human embryonic stem cell-derived lung organoids. Stem Cell Res Ther. 2020 Oct 23; 11(1): p. 448.

SUPPLEMENTAL TABLE

SEQUENCES OF THE OLIGONUCLEOTIDES EMPLOYED IN THIS STUDY	
18S	5'- GTAACCCGTTGAACCCCATT
	5'- CCATCCAATCGGTAGTAGCG
PDPN (marker of ATI cells)	5'- AGGAGAGCAACAACTCAACGGGA
	5'- TTCTGCCAGGACCCAGAGC
SFTPA (marker of ATII cells)	5'-GTGCGAAGTGAAGGACGTTTGTG
	5'-TTTGAGACCATCTCTCCCGTCCC
SFTPB(marker of ATII cells)	5'-TCTGAGTGCCACCTCTGCATGT
	5'-TGGAGCATTGCCTGTGGTATGG
SFTPC(marker of ATII cells)	5'-CCTTCTTATCGTGGTGGTGGTGGT
	5'-TCTCCGTGTGTTTCTGGCTCATGT
SFTPD(marker of ATII cells)	5'-TGACTGATTCCAAGACAGAGGGCA
	5'-TCCACAAGCCCTGTCATTCCACTT
IL6 (INTERLEUKIN 6)	5'- TGGCTGCAGGACATGACAACT
	5'-ACAATCTGAGGTGCCCATGCT
IL8 (INTERLEUKIN 8, CXC8)	5'-GCAGTTTTGCCAAGGAGTGTC
	5'-TTTCTGTGTTGGCGCAGTGTG
TNFα (TNF)	5'- GCCCATGTTGTAGCAAACCCT
	5'-TCGGCAAAGTCGAGATAGTCG
TLR2	5'- CTTCACTCAGGAGCAGCAAGCA
	5'- ACACCAGTGCTGTCCTGTGACA
CXCL5	5'- CAGACCACGCAAGGAGTTCATC
	5'- TTCCTTCCCGTTCTTCAGGGAG
CCL20	5'- AAGTTGTCTGTGTGCGCAAATCC
	5'- CCATTCCAGAAAAGCCACAGTTTT
STING1 (TMEM173)	5'- CCTGAGTCTCAGAACAACTGCC,
	5'- GGTCTTCAAGCTGCCCACAGTA
